# Supplementary material for: A Study of Metabolites from Basidiomycota and Their Activities against Pseudomonas aeruginosa
Source: Antibiotics (Basel). 2024 Apr 3;13(4):326. doi: 10.3390/antibiotics13040326 (PMC11047493; doi:10.3390/antibiotics13040326)
Supplement: Supplementary file 1 [file antibiotics-13-00326-s001.zip › antibiotics-2920118-supplementary.pdf]

A study of metabolites from Basidiomycota and their activity against Pseudomonas aeruginosa

M. Clericuzio; G. Novello, M. Bivona; E. Gamalero; E. Bona; A. Caramaschi; N. Massa; A. Asteggiano, C. Medana

SUPPLEMENTARY MATERIAL

Contents

Figure S1. Partial (low field) ^{1}H NMR spectrum of compound **1a**.

Figure S2. Partial (high field) ^{1}H NMR spectrum of compound **1a**.

Figure S3. Partial (low field) ^{13}C NMR spectrum of compound **1a**.

Figure S4. Partial (high field) ^{13}C NMR spectrum of compound **1a**.

Figure S5. ^{1}H NMR spectrum of compound **2a**.

Figure S6. ^{13}C NMR spectrum of compound **2a**.

Figure S7. ^{1}H NMR spectrum of compound **2b**.

Figure S8. ^{1}H NMR spectrum of compound **3**.

Figure S9. Partial (low field) HSQC NMR spectrum of compound **3**.

Figure S10. Partial (high field) HSQC NMR spectrum of compound **3**.

Figure S11. ^{1}H NMR spectrum of compound **4**. Peaks at 7.55 and 6.25 ppm are assigned to compound **5** (13-KODE), present in approximately 12% molar.

Figure S12. ^{13}C NMR spectrum of compound **4**.

Figure S13. Partial ^{1}H COSY NMR spectrum of compound **4**. The correlation between peaks at 7.55 and 6.25 ppm confirms the structure of compound **5**.

Figure S14. ^{1}H NMR spectrum of compound **6**.

Figure S15. ^{13}C NMR spectrum of compound **6**.

Figure S16. ^{1}H NMR spectrum of fraction 16F/2.

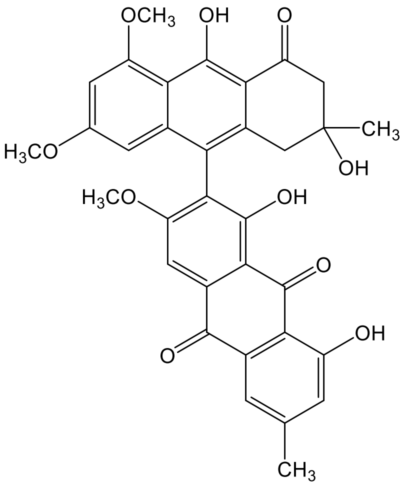

1a

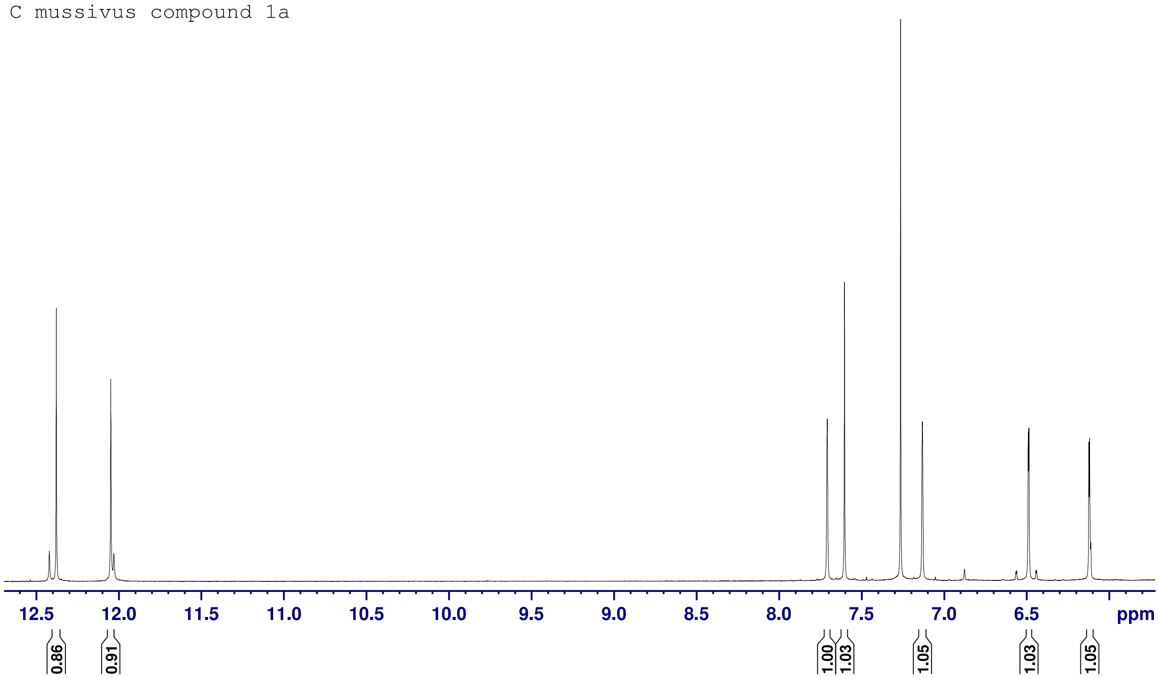

Figure S1. Partial (low field) ^{1}H NMR spectrum of compound **1a**.

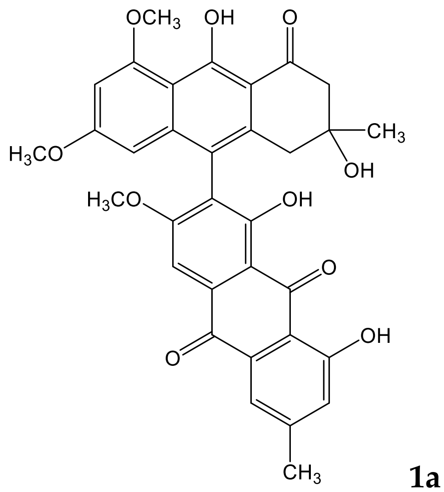

1a

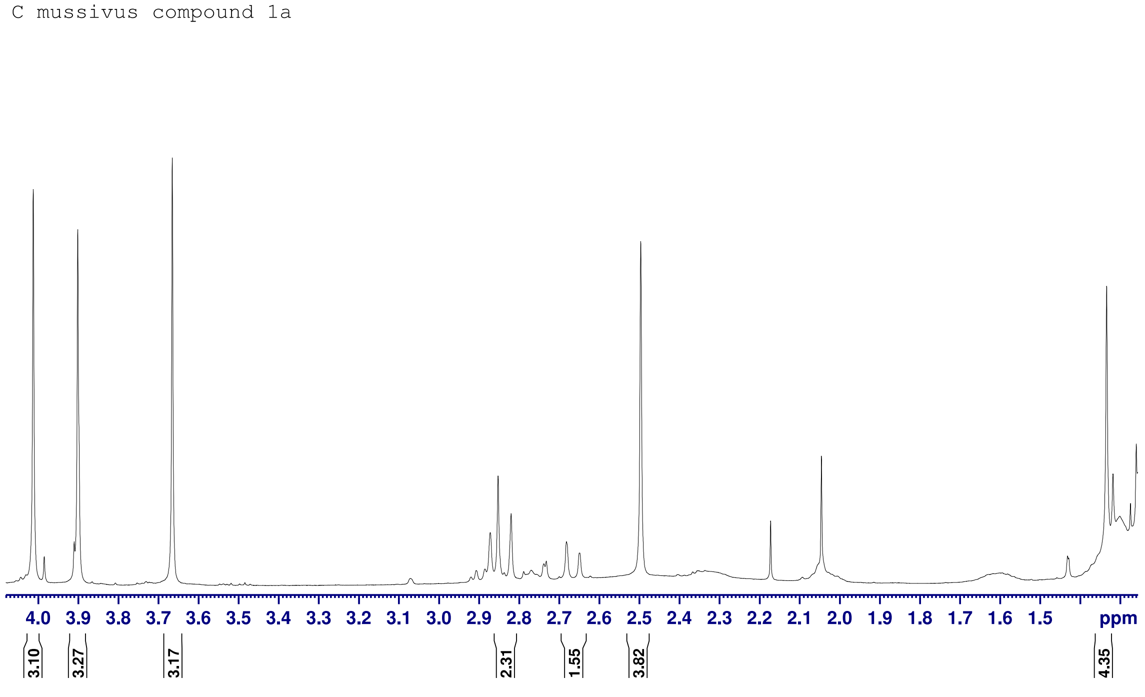

Figure S2. Partial (high field) ^{1}H NMR spectrum of compound **1a**.

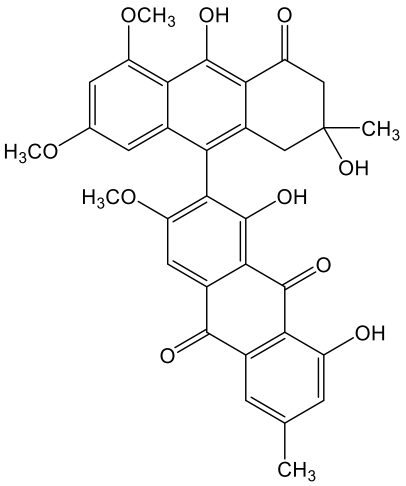

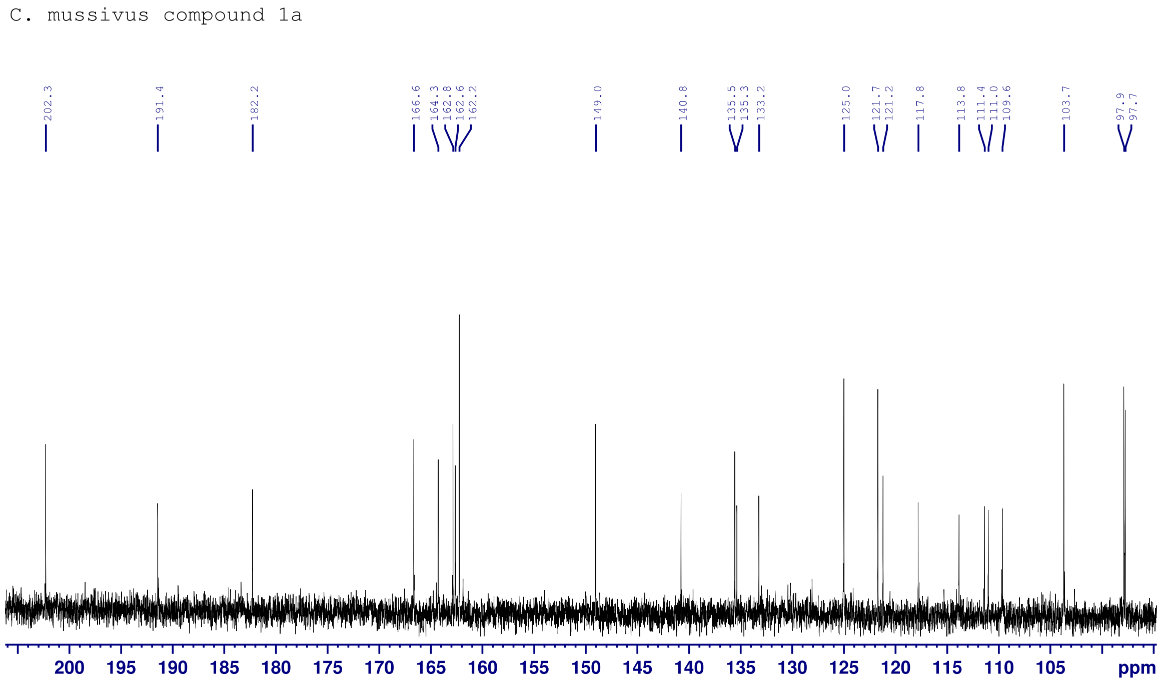

Figure S3. Partial (low field) ^{13}C NMR spectrum of compound **1a**.

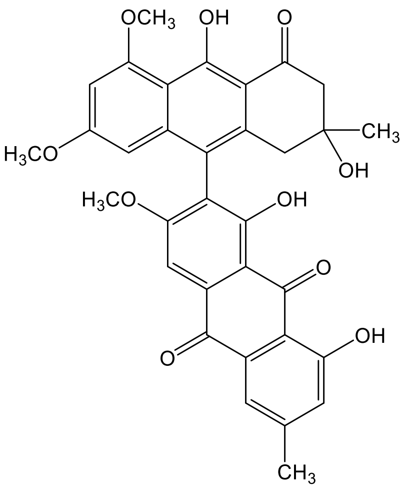

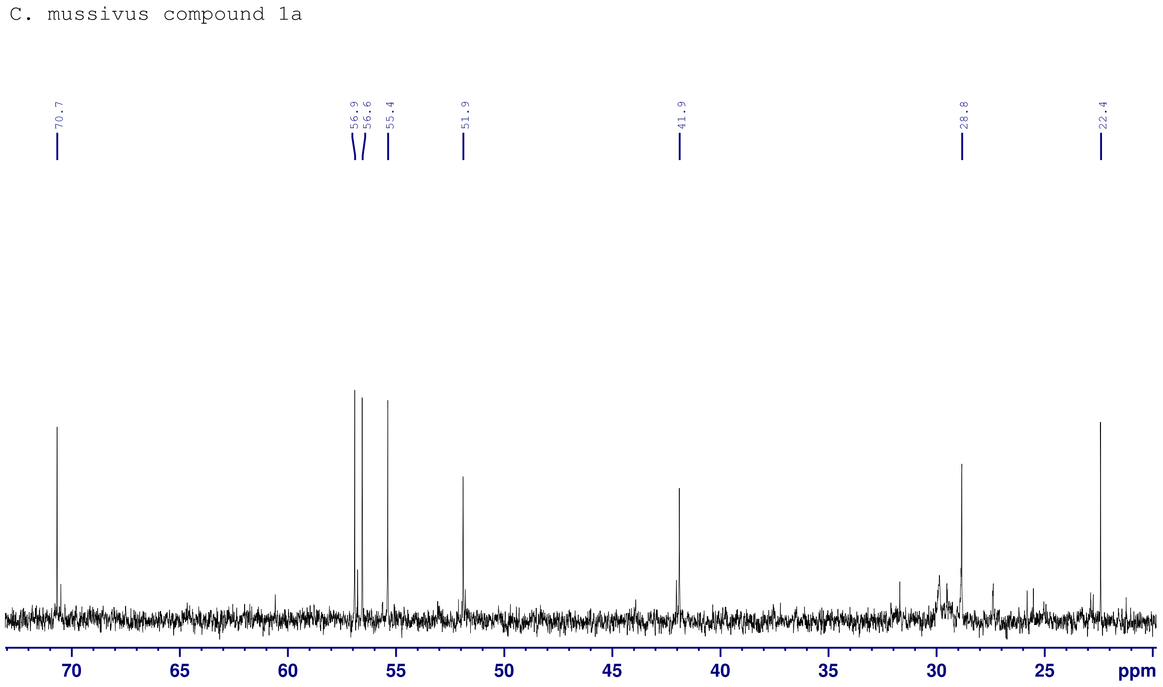

Figure S4. Partial (high field) ^{13}C NMR spectrum of compound **1a**.

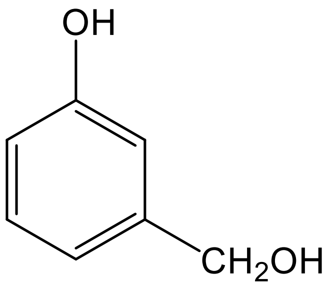

2a

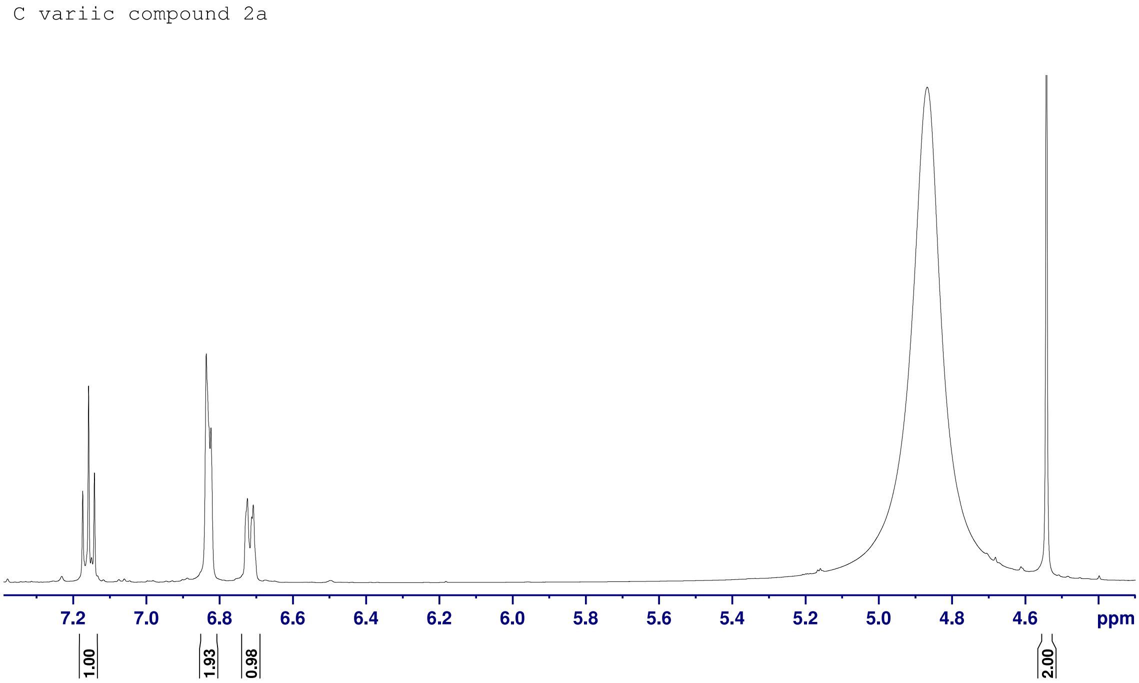

Figure S5. ^{1}H NMR spectrum of compound **2a**.

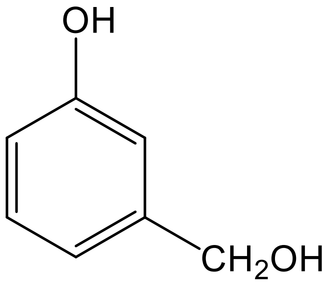

2a

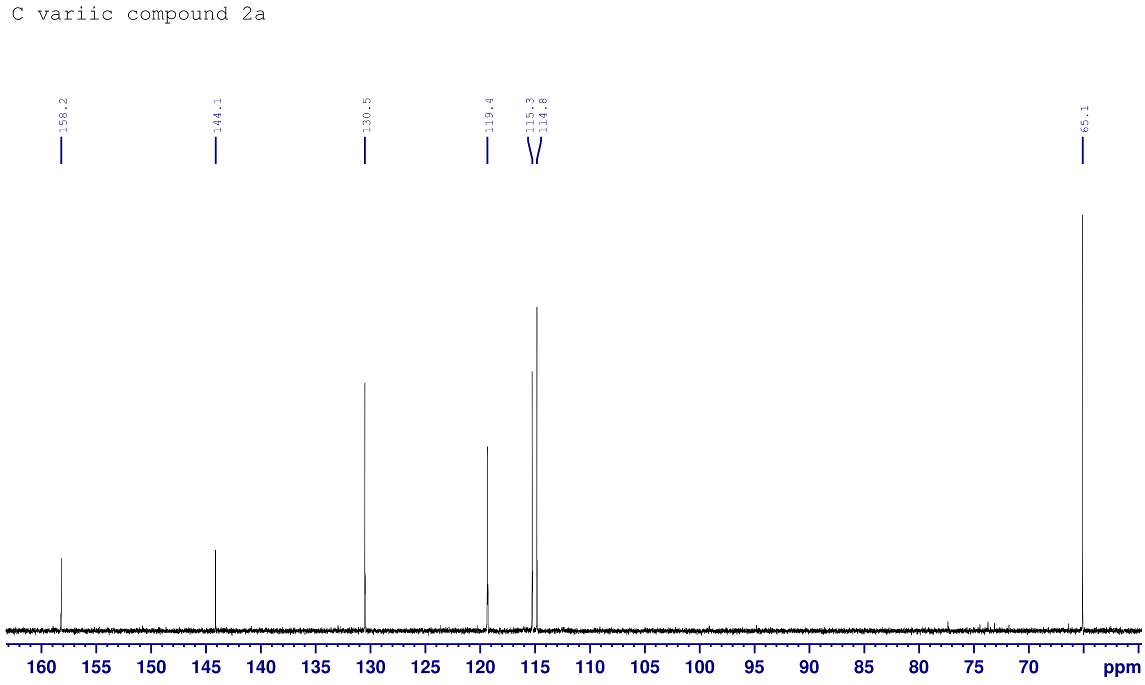

Figure S6. ^{13}C NMR spectrum of compound **2a**.

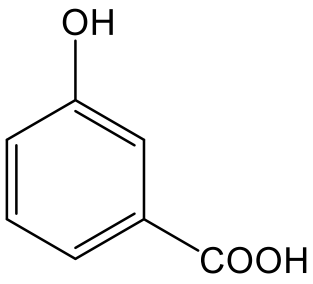

2b

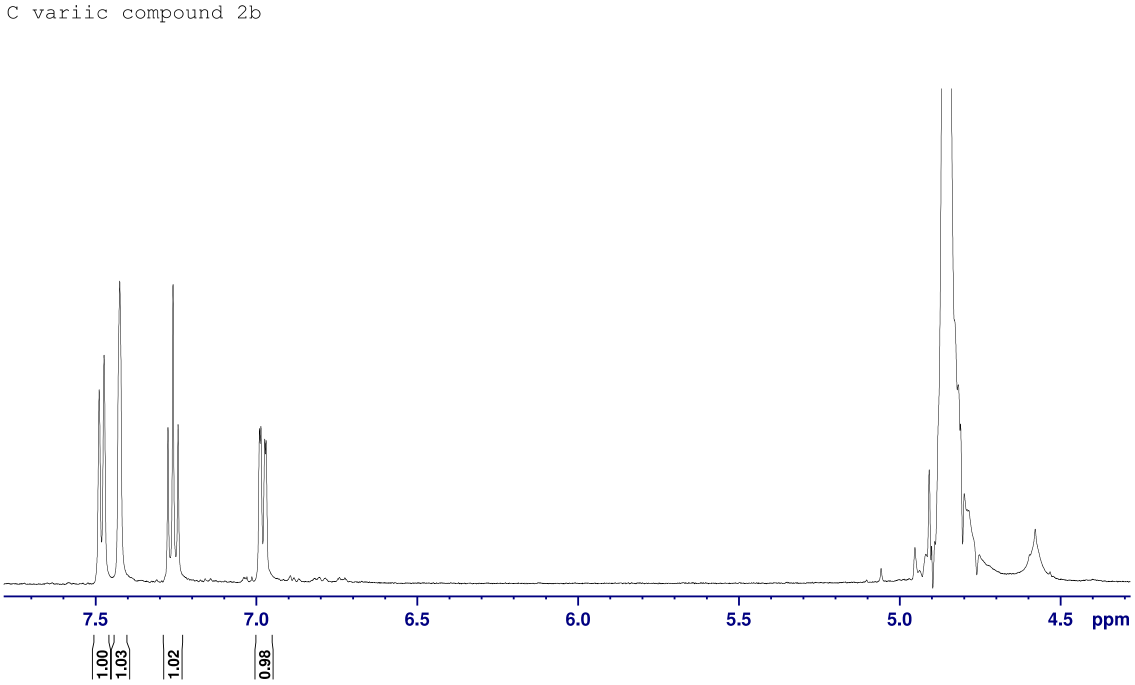

Figure S7. ^{1}H NMR spectrum of compound **2b**.

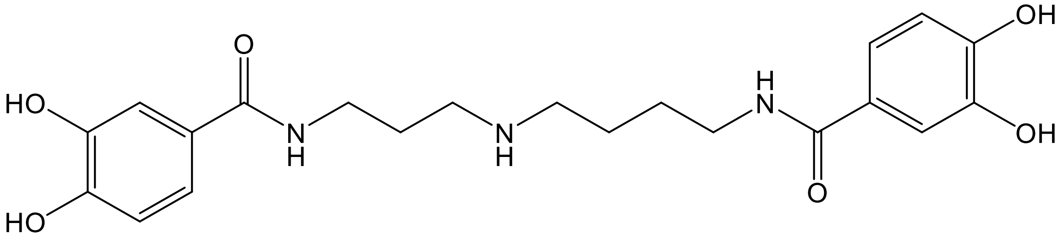

3

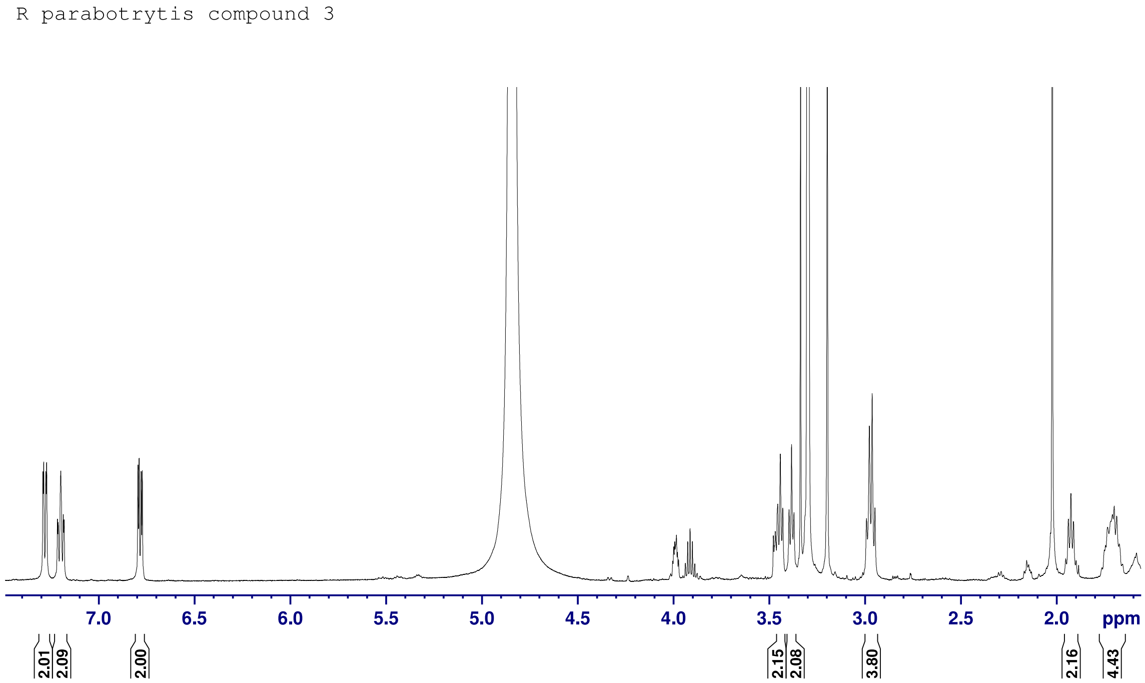

Figure S8. ^{1}H NMR spectrum of compound **3**.

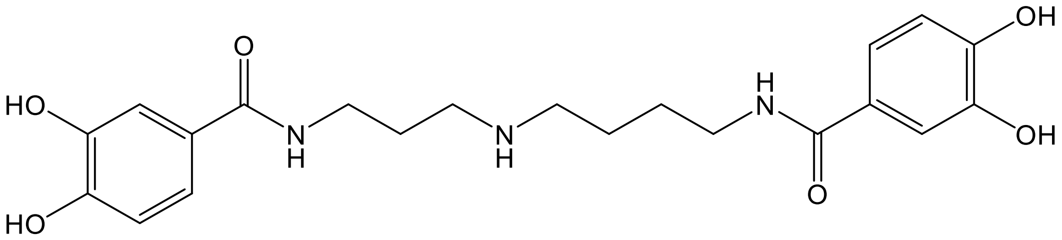

3

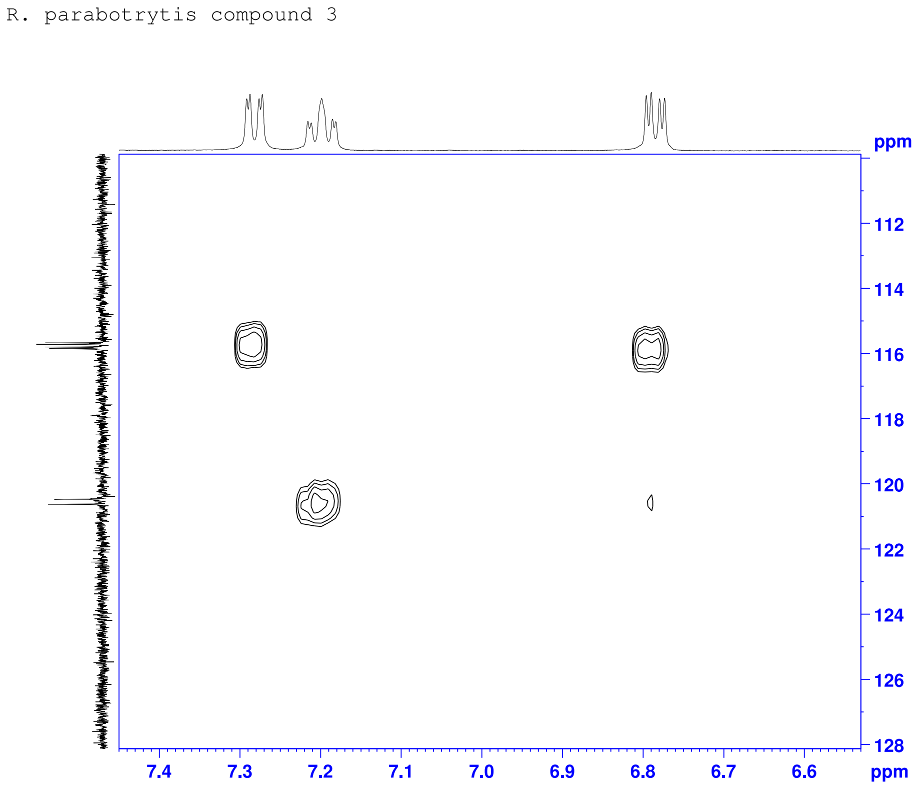

Figure S9. Partial (low field) HSQC NMR spectrum of compound **3**.

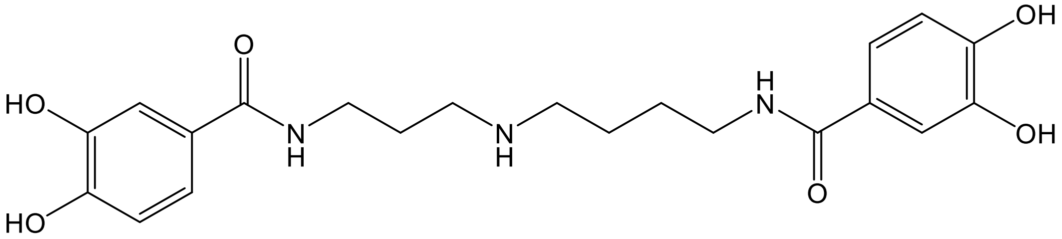

3

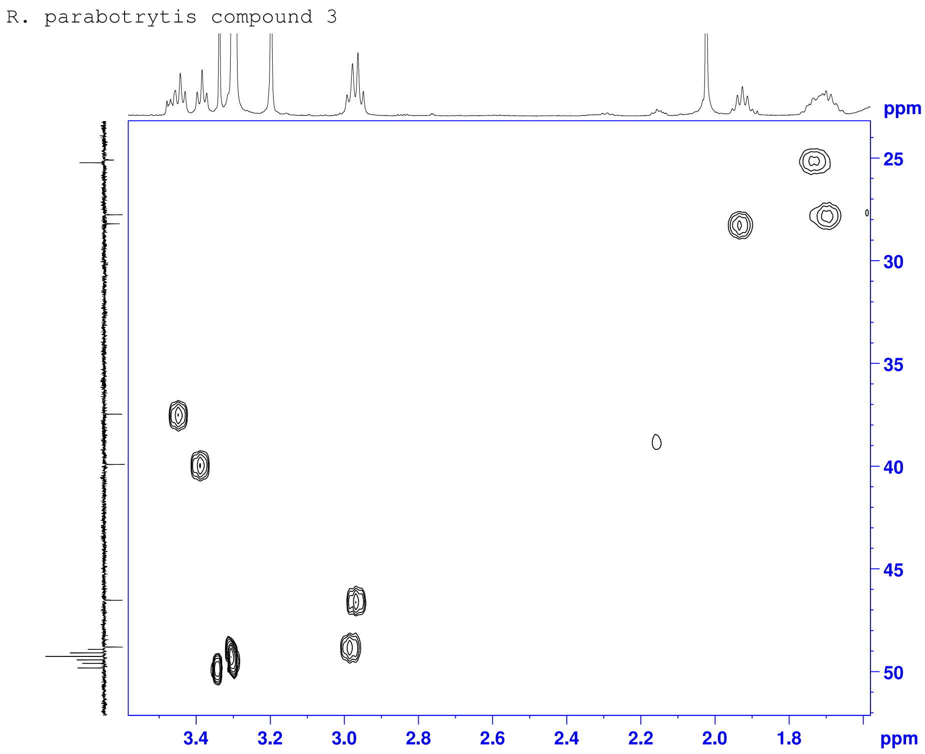

Figure S10. Partial (high field) HSQC NMR spectrum of compound **3**.

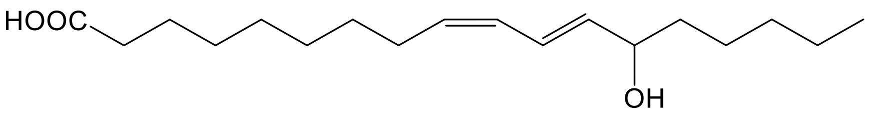

4

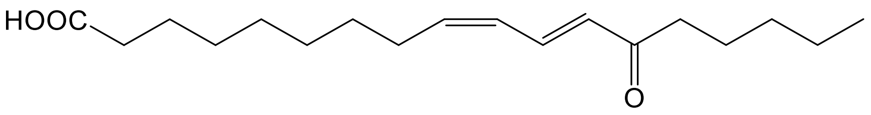

5

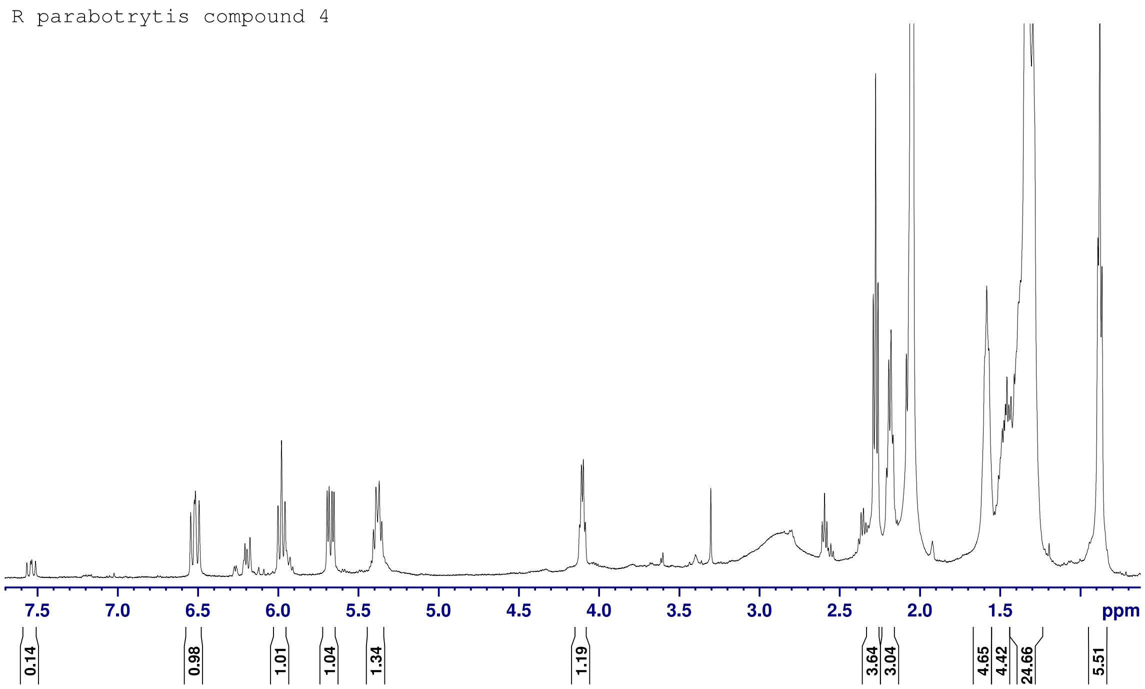

Figure S11. ^{1}H NMR spectrum of compound **4**. Peaks at 7.55 and 6.25 ppm are assigned to compound **5** (13-KODE), present in approximately 12% molar.

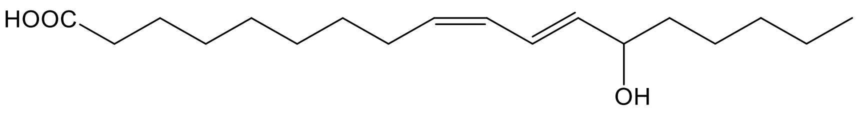

4

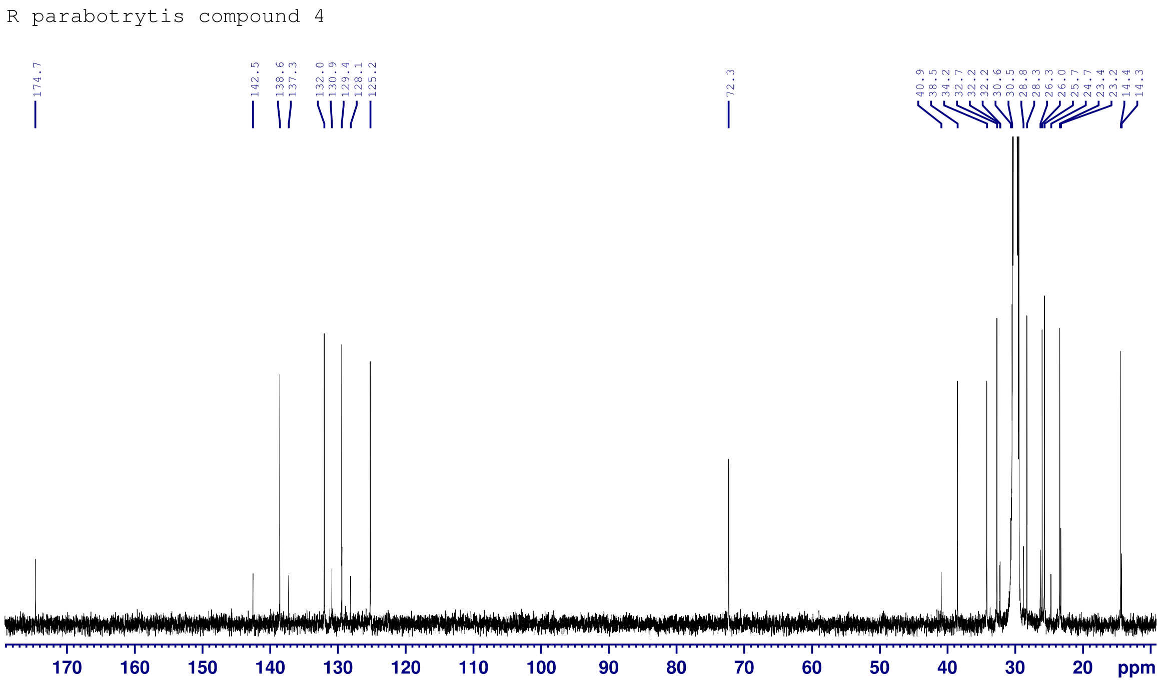

Figure S12. ^{13}C NMR spectrum of compound **4**.

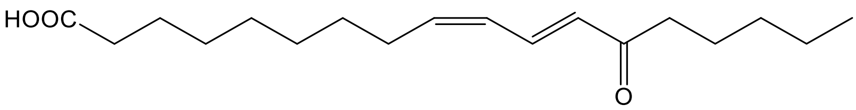

5

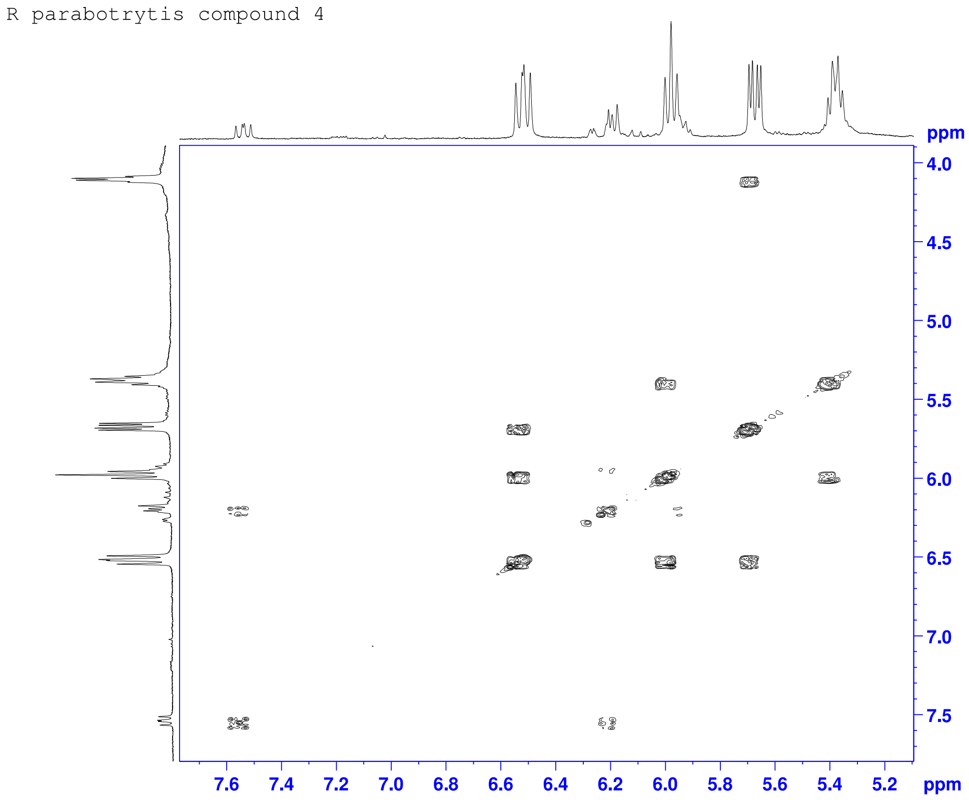

Figure S13. Partial ^{1}H COSY NMR spectrum of compound **5** (in mixture with **4**). The correlation between peaks at 7.55 and 6.25 ppm confirms the structure of compound **5**.

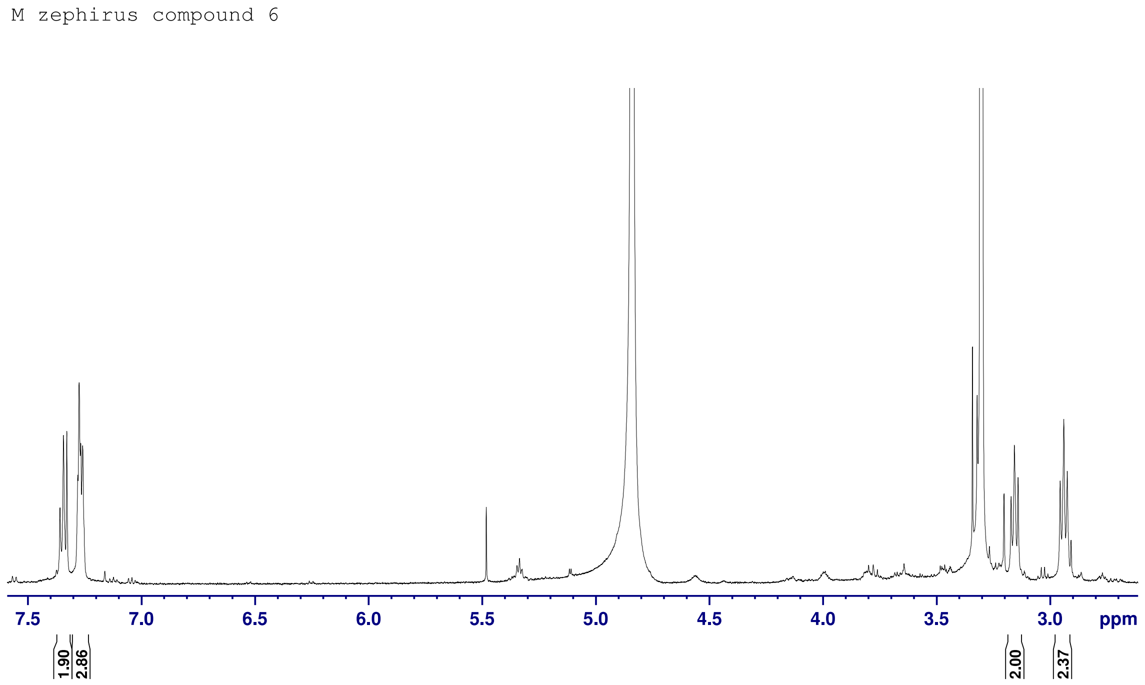

Figure S14. ^{1}H NMR spectrum of compound **6**.

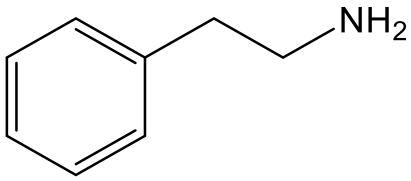

6

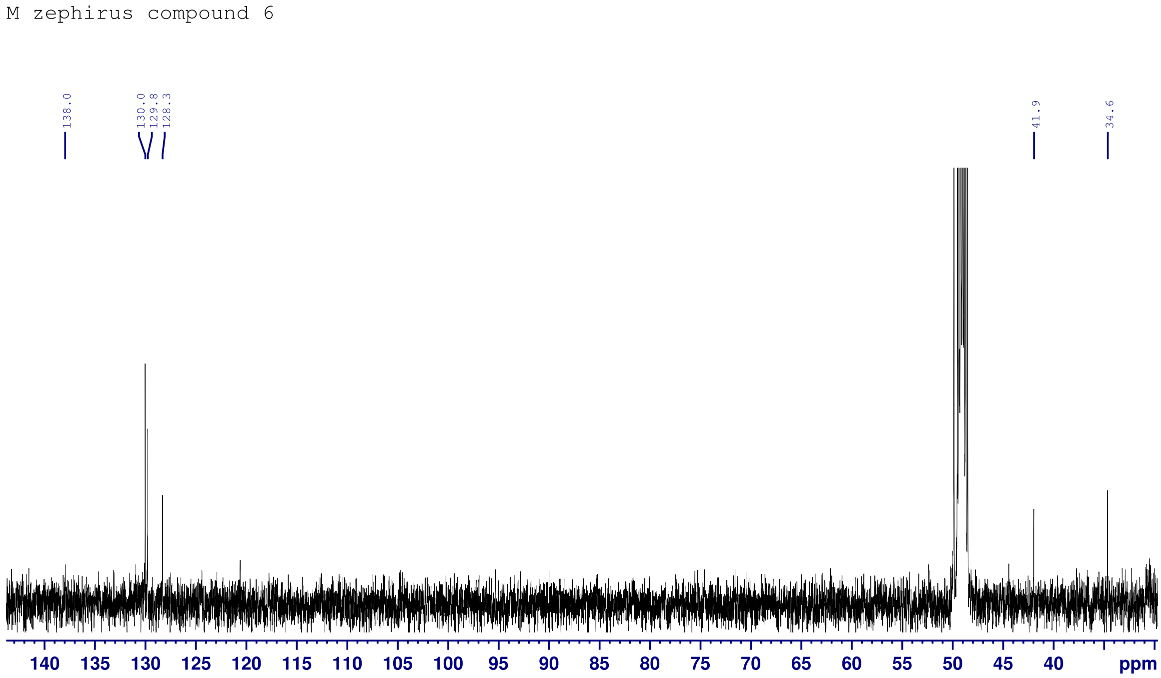

Figure S15. ^{13}C NMR spectrum of compound **6**.

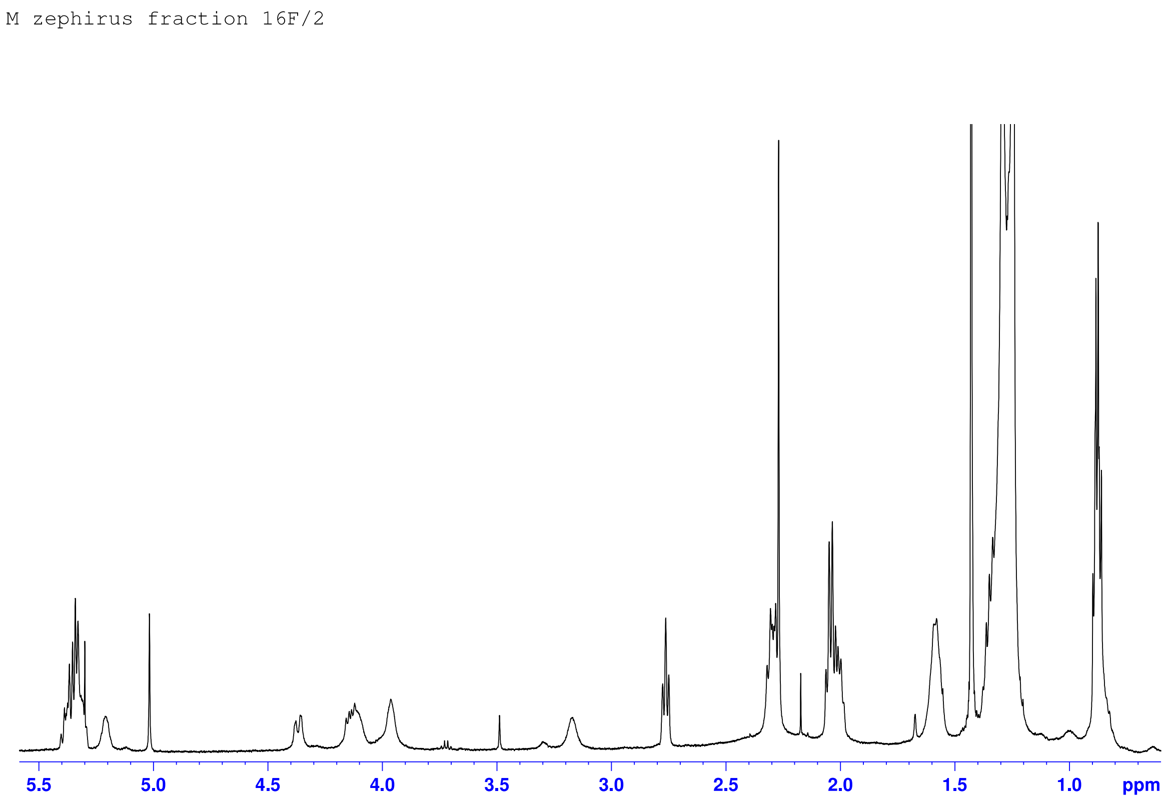

Figure S16. ^{1}H NMR spectrum of fraction 16F/2.
